# Enteroviruses in Patients with Acute Encephalitis, Uttar Pradesh, India

**DOI:** 10.3201/eid1502.080865

**Published:** 2009-02

**Authors:** Gajanan N. Sapkal, Vijay P. Bondre, Pradip V. Fulmali, Pooja Patil, Gopalkrishna, Vipul Dadhania, Vijay M. Ayachit, Daya Gangale, K.P. Kushwaha, A.K. Rathi, Shobha D. Chitambar, Akhilesh Chandra Mishra, Milind M. Gore

**Affiliations:** National Institute of Virology, Pune, India (G.N. Sapkal, V.P. Bondre, P.V. Fulmali, P. Patil, V. Gopalkrishna, V. Dadhania, V.M. Ayachit, D. Gangale, S.D. Chitambar, A.C. Mishra, M.M. Gore); Baba Raghav Das Medical College, Gorakhpur, India (K.P Kushwaha, A.K. Rathi)

**Keywords:** Human enterovirus 76, viral encephalitis, Japanese encephalitis virus, Uttar Pradesh, India, dispatch

## Abstract

An outbreak of viral encephalitis occurred in northern India in 2006. Attempts to identify an etiologic agent in cerebrospinal fluid by using reverse transcription–PCR showed positivity to enterovirus (EV) in 66 (21.6%) of 306 patients. Sequencing and phylogenetic analyses of PCR products from 59 (89.3%) of 66 specimens showed similarity with EV-89 and EV-76 sequences.

Acute viral encephalitis is caused by a wide range of viruses and can occur either in sporadic episodes or in outbreaks. Viral etiologic agents that have been identified as causing encephalitis include herpesvirus, enterovirus, alphavirus, influenza A virus, rabies virus, HIV, flavivirus, and Chandipura (CHP) virus (*1*,*2*). An outbreak of viral encephalitis was reported from April through October 2006 from predominantly Gorakhpur and 5 adjoining districts of eastern Uttar Pradesh (Maharajganj, Kushinagar, Sant Kabir Nagar, Siddharthnagar, and Deoria) and 2 adjoining districts of Bihar (Gopalganj and West Champaran), locations where Japanese encephalitis (JE) is known to be endemic in India. According to state government health services records, 1,912 cases of viral encephalitis occurred in these areas, and 411 (21.5%) patients died. From August through September 2006, we investigated 306 patients admitted with encephalitis to Baba Raghav Das Medical College in Gorakhpur, Uttar Pradesh. The patients represented all 8 districts of eastern Uttar Pradesh. ELISA and reverse transcription–PCR (RT-PCR) performed on the patients’ cerebrospinal fluid (CSF) samples identified 40 (13.1%) of 306 specimens as positive for JE virus (*3*). Laboratory tests were negative for alphavirus and CHP virus, and the etiologic agent in a large number of cases was unidentified.

Enteroviruses (EVs) cause a wide variety of diseases that range from nonspecific viral illness to mild infections of herpangina and hand, foot, and mouth disease to potentially serious diseases such as myopericarditis, meningitis, myelitis, and neonatal sepsis. EVs are also etiologic agents of encephalitis outbreaks in humans (*4*). These viruses comprise more than 90 serotypes, and most are known to cause human infections. We focused on the detection, isolation, and molecular characterization of EVs in 306 patients from eastern Uttar Pradesh.

## The Study

A total of 850 specimens collected from 306 patients who had encephalitis included 306 CSF specimens, 304 blood samples, 120 throat swabs, and 120 rectal swabs. All samples were stored at –20^o^C before being transported for analysis and thereafter were stored at –70^o^C at the National Institute of Virology in Pune, India. Laboratory tests conducted by state government health services of Uttar Pradesh were negative for bacteria and malaria. According to standard protocol (*2*), virus isolation was attempted in human rhabdosarcoma (RD) and in baby hamster kidney (BHK) cell lines.

Separate aliquots were processed in 2 laboratories to maintain quality control and monitor possible contamination during PCR processing. Viral nucleic acids were extracted by using viral RNA mini kits (QIAamp, Qiagen, Hilden, Germany). RT-PCR was performed for EV by using 5′ noncoding region (NCR)–specific primers, as has been described (*5*,*6*). Genotyping was conducted by using RT-PCR of virion protein (VP) 1/2A and VP1 regions and sequencing (*7*,*8*). Table 1 describes the locations and sequences of the primers used in the assays.

**Table 1:** Primers used for PCR and sequencing for enterovirus isolates from encephalitis patients, Uttar Pradesh, India, 2006*

Serial no.	Region	Location	Primer sequence (5′ → 3′)	Product size, bp
1	5’ NCR	64–84	CGGTACCTTTGTACGCCTGT	537
2	5’ NCR	601–582	ATTGTCACCATAAGCAGCCA	537
3	5’ NCR	166–186	CAAGCACTTCTGTTTCCCCGG	400
4	5’ NCR	566–546	GAAACACGGACACCCAAAGTA	400
5	VP1/2A	EV-012 (2917–2936)	ATGTAYGTICCICCIGGIGG	457
6	VP1/2A	EV-040 (2917–2936)	ATGTAYRTICCIMCIGGIGC	457
7	VP1/2A	EV-011 (3374–3355)	GCICCIGAYTGITGICCRAA	457
8	VP1 cDNA	AN32 (3009–3002)	GTYTGCCA	NA
9	VP1 cDNA	AN33 (3009–3002)	GAYTGCCA	NA
10	VP1 cDNA	AN34 (3111–3104)	CCRTCRTA	NA
11	VP1 cDNA	AN35 (3009–3002)	RCTYTGCCA	NA
12	VP3	224(1977–1996)	GCIATGYTIGGIACICAYRT	762
13	VP1	222 (2969–2951)	CICCIGGIGGIAYRWACAT	762
14	VP1	AN89 (2602–2627)	CCAGCACTGACAGCAGYNGARAYNGG	348–393
15	VP1	AN88 (2977–2951)	TACTGGACCACCTGGNGGNAYRWACAT	348–393

*NCR, noncoding region of viral genome; VP, virion protein; NA, not applicable.

PCR products were purified by using a Gel Extraction Kit (QIAquick, Qiagen). Both strands were sequenced by using BigDye Terminator Cycle Sequencing Ready Reaction Kit (Applied Biosystems, Carlsbad, CA, USA) in ABI PRISM 3130 XL Genetic Analyser (Applied Biosystems). MEGA 3.1 software generated the phylogenetic tree by using the neighbor-joining algorithm and Kimura 2–parameter distance model and applying a bootstrap test that used 1,000 bootstrap replications (*9*).

Patient age ranged from <1 month to 15 years. Clinical histories available for 253 of the 306 patients showed fever and altered sensorium in 100.0%, hepatomegaly in 70 (27.8%), splenomegaly in 49 (19.4%), and meningeal signs in 35 (13.9%) of the 253 patients.

Specimens available in sufficient quantity were inoculated into RD and BHK cell lines. Specimens that were adequate for isolation included 85 of 306 CSF specimens, 18 of 304 serum samples, 19 of 120 rectal swabs, and 19 of 120 throat swabs. Cytopathic effect was observed in cell cultures inoculated with 4 CSF specimens, 2 rectal swabs, 2 throat swabs, and 1 serum sample. Electron microscopic examination of cultures infected with 2 CSF samples showed picornavirus-like particles 25–27 nm in diameter. Attempts to detect EV RNA in the isolates and clinical specimens used nested RT-PCR in 5′ NCR. Eight of 9 cultures showed amplicons of 407 bp. Sequences of amplicons from 3 CSF specimens and 2 rectal swabs showed 97.2%–98.9% homology with EV-89 (i.e., the strain named BANoo-10359, GenBank accession no. AY697459) and 95.7%–96.9% homology with EV-76 (FRA91-10369, GenBank accession no. AY697458). Sequences from 1 isolate from a CSF specimen and 1 isolate from a rectal swab showed 100.0% homology with coxsackie virus B3 (CV-B3) strain 20. One isolate from serum showed 98.3% homology with coxsackie virus B1 (CV-B1) strain SAMP2.17.

**Figure 1:**
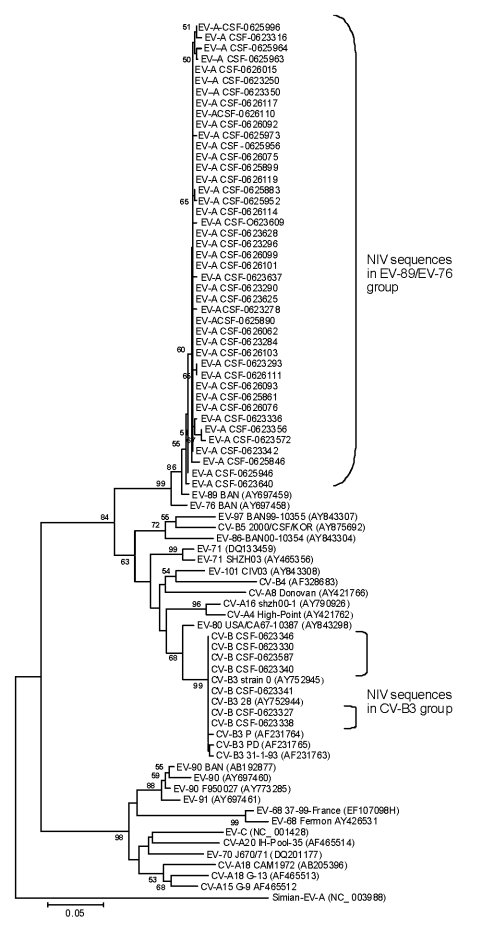
Phylogenetic tree based on partial 5’ noncoding region sequences of enterovirus (EV) genome detected in cerebrospinal fluid samples from encephalitis patients. Specimens are identified by repository serial numbers obtained from the National Institute of Virology (NIV), Pune, India. GenBank accession nos. EU672893–EU762967 indicate the nucleotide sequences of EV strains of the present study. Scale bar indicates nucleotide substitutions per site. EV, enterovirus; CSF, cerebrospinal fluid; CV-A, coxsackie virus A; CV-B, coxsackie virus B; HEV, human enterovirus.

Sixty-six (21.5%) of 306 CSF specimens, 7 (6.4%) of 110 rectal swabs, 4 (3.7%) of 110 throat swabs, and 1 (5.5%) of 18 serum samples showed amplification in 5′ NCR of the EV genome. Sequences of 64 of 78 (82.0%) PCR products (59 from CSF specimens, 4 from rectal swabs, and 1 from a throat swab) showed 97.2%–98.9% and 95.7%–96.9% homology with EV-89 and EV-76, respectively. Ten (12.8%) products (7 from CSF, 2 from rectal swabs, and 1 from serum) showed 99.3%–100.0% homology with CV-B3 (Figure 1). Three PCR products, each derived from a throat swab, showed 93.3%–96.6% homology with coxsackie virus A (CV-A), echovirus 11, and echovirus 30, respectively. PCR products from a rectal swab showed 96.3% homology with CV-B1. Multiple specimen positivity was noted in 6 patients who tested positive for EV RNA.

**Figure 2:**
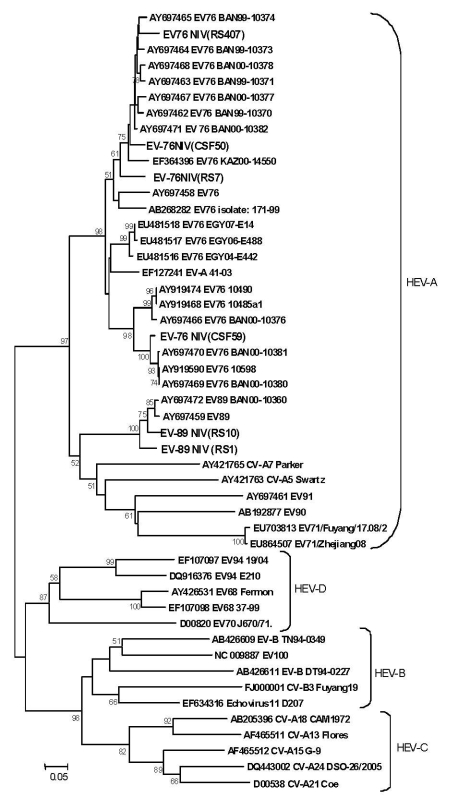
Phylogenetic tree based on partial virion protein 1 (VP1) sequences (2602–2977) detected in enterovirus (EV) isolates and clinical specimens from encephalitis patients. GenBank accession nos. indicate the nucleotide sequences of EV strains of the present study. Scale bar indicates nucleotide substitutions per site. EV, enterovirus; CV-A, coxsackie virus A; CV-B, coxsackie virus B; HEV, human enterovirus; NIV, National Institute of Virology, Pune, India.

Isolates from 2 of 5 cell cultures, 2 of 59 CSF specimens, and 1 of 4 rectal swabs contained EV-76. Two of 4 rectal swabs were characterized as EV-89 on the basis of partial VP1/2A (2917–3374) or VP1 (2602–2977) gene sequences. Phylogenetic analysis revealed 92.7%–97.7% homology with Bangladesh EV-76 strains (GenBank accession nos. AY697463, AY697464, AY697471, AY697469, AY697462, and AY697468) and 93.6%–94.5% homology with EV-89 strain (GenBank accession no. AY697459) (Figure 2). Within EV-76 and EV-89 strains of the study, homology ranged from 81.2% to 91.3%. Attempts to amplify VP1/2A or VP1 regions of EV RNA detected in most clinical specimens failed despite the use of sensitive primer pairs that have been discussed recently (*10*).

Table 2 describes details of clinical findings in the subsets of EV-positive and EV-negative specimens of the patients for whom clinical histories were available. Further, hepatomegaly and splenomegaly appeared to be proportionately higher in patients with enteroviral infections than in patients whose specimens were negative for EV and JE virus.

**Table 2:** Clinical features of encephalitis patients with and without EV infections, Upper Pradesh, India, 2006*

Feature	No. (%) EV positive	No. (%) EV negative
Fever	51 (100.0)	202 (100.0)
Altered sensorium	51 (100.0)	202 (100.0)
Hepatomegaly	13 (25.49)	21 (10.3)
Splenomegaly	13 (25.49)	32 (15.8)
Brisk DTR	4 (8.51)†	21 (10.3)
Meningeal signs	6 (11.7)	20 (9.9)
Total no. of patients	51	202

*EV, enterovirus; DTR, deep tendon reflex. †Denominator is 47 because these data were unavailable for 4 patients.

## Conclusions

The viral RNA detected in CSF samples from patients hospitalized with encephalitis in Uttar Pradesh showed close identity with the EV-89 and EV-76 that recently were reported as an unusual group classified genetically as group A EV (EV-A) (*10*). Presence of the virus was also confirmed by its isolation and typing. Human EV-76 was detected in isolates in 1 rectal swab and 2 CSF specimens, and human EV-89 was detected in 2 rectal swabs by using amplification of VP1/2A or VP1 regions. Sequence analysis showed nt homology of 92.7%–97.7% with Bangladesh EV-76 and EV-89 strains recovered from patients with acute flaccid paralysis (AFP). The failure of amplification of typing regions in most specimens may be due to a low viral load.

EVs are known to cause severe neurologic diseases ranging from AFP to encephalitis (*11*). In recent years, Southeast Asian countries have reported outbreaks of encephalitis caused by EV-71 (*12*,*13*). During AFP surveillance activities, Bangladesh strains were isolated from stool specimens (*14*). AFP patients infected with echoviruses and coxsackie B viruses also have been detected in India (*15*). Isolation of EV from clinical specimens collected from children with encephalitis in the present study indicates viable virus. Detection of EV-89/76 RNA in the CSF of ≈20% of the patients suggests the association of these viruses with encephalitis. Also, in 10 (3.3%) of 306 patients, co-infections of JE virus and EV were detected. Further studies are needed to understand the relative contributions of these viruses in causing sporadic and outbreak infections of encephalitis.

Accumulation of water in a saucer-shaped landscape (*terai*) and extensive rice cultivation in eastern Uttar Pradesh and adjoining regions favor the growth of vector mosquito populations and waterborne pathogens. Though the source of infection in the present study is unclear, the data warrant active surveillance of encephalitis cases. Inadequate hygiene and the unsanitary conditions that prevail in the study region may encourage the spread of EV infections in the community. Studies conducted on environmental samples may provide clues related to the dynamics of EV infections in humans.

## References

[1] Kennedy PG. Viral encephalitis: causes, differential diagnosis, and management. J Neurol Neurosurg Psychiatry. 2004;75(Suppl 1):i10–5. 10.1136/jnnp.2003.034280 14978145 PMC1765650

[2] Rao BL, Basu A, Wairagkar NS, Gore MM, Arankalle VA, Thakare JP, A large outbreak of acute encephalitis with high fatality rate in children in Andhra Pradesh, India, in 2003, associated with Chandipura virus. Lancet. 2004;364:869–74. 10.1016/S0140-6736(04)16982-1 15351194 PMC7137741

[3] Sapkal GN, Wairagkar NS, Ayachit VM, Bondre VP, Gore MM. Detection and isolation of Japanese encephalitis virus from blood clots collected during the acute phase of infection. Am J Trop Med Hyg. 2007;77:1139–45. 18165537

[4] Pallansch MA, Roos RP. Enteroviruses: polioviruses, coxsackieviruses, echoviruses, and newer enteroviruses. In: Fields virology, 5th ed. Knipe DM, Howley PM, Griffin DE, Lamb RA, Martin MA, Roizman B, et al., editors. Philadelphia: Lippincott Williams & Wilkins; 2006. p. 839–94.

[5] Zoll GJ, Melchers WJ, Kopecka H, Jambroes G, van der Poel HJ, Galama JM. General primer-mediated polymerase chain reaction for detection of enteroviruses: application for diagnostic routine and persistent infections. J Clin Microbiol. 1992;30:160–5. 1370845 10.1128/jcm.30.1.160-165.1992 PMC265013

[6] Puig M, Jofre J, Lucena F, Allard A, Wadell G, Girones R. Detection of adenoviruses and enteroviruses in polluted waters by nested PCR amplification. Appl Environ Microbiol. 1994;60:2963–70. 8085832 10.1128/aem.60.8.2963-2970.1994 PMC201750

[7] Oberste MS, Maher K. Kilpatrick. DR, Flemister MR, Brown BA, Pallansch MA. Typing of human enteroviruses by partial sequencing of VP1. J Clin Microbiol. 1999;37:1288–93. 10203472 10.1128/jcm.37.5.1288-1293.1999 PMC84754

[8] Nix WA, Oberste MS, Pallansch MA. Sensitive, seminested PCR amplification of VP1 sequences for direct identification of all enterovirus serotypes from original clinical specimens. J Clin Microbiol. 2006;44:2698–704. 10.1128/JCM.00542-06 16891480 PMC1594621

[9] Kumar S, Tamura K, Jakobsen IB, Nei M. MEGA2: molecular evolutionary genetics analysis software. Bioinformatics. 2001;17:1244–5. 10.1093/bioinformatics/17.12.1244 11751241

[10] Oberste MS, Maher K, Michele SM, Bellot G, Uddin M, Pallansch MA. Enteroviruses 76, 89, 90 and 91 represent a novel group within the species Human enterovirus A. J Gen Virol. 2005;86:445–51. 10.1099/vir.0.80475-0 15659764

[11] Wildin S, Chonmaitree T. The importance of the virology laboratory in the diagnosis and management of viral meningitis. Am J Dis Child. 1987;141:454–7. 3031978 10.1001/archpedi.1987.04460040112030

[12] Hayward JC, Gillespie SM, Kaplan KM, Packer R, Pallansch M, Plotkin S, Outbreak of poliomyelitis-like paralysis associated with enterovirus 71. Pediatr Infect Dis J. 1989;8:611–6. 10.1097/00006454-198909000-00009 2797956

[13] Kehle J, Roth B, Metzger C, Pfitzner A, Enders G. Molecular characterization of an enterovirus 71 causing neurological disease in Germany. J Neurovirol. 2003;9:126–8. 10.1080/713831340 12587076

[14] Oberste MS, Penaranda S, Maher K, Pallansch MA. Complete genome sequences of all members of the species *Human enterovirus A.* J Gen Virol. 2004;85:1597–607. 10.1099/vir.0.79789-0 15166444

[15] Kapoor A, Ayyagari A, Dhole TN. Non-polio enteroviruses in acute flaccid paralysis. Indian J Pediatr. 2001;68:927–9. 10.1007/BF02722583 11758127

